# COVID-19 in Relation to Polypharmacy and Immunization (2020–2024)

**DOI:** 10.3390/v16101533

**Published:** 2024-09-27

**Authors:** Anna Puigdellívol-Sánchez, Marta Juanes-González, Ana Calderón-Valdiviezo, Roger Valls-Foix, Marta González-Salvador, Celia Lozano-Paz, Josep Vidal-Alaball

**Affiliations:** 1 Medicina de Família, CAP Anton de Borja Centre Universitari, c/ Marconi-Cantonada Edison s/n, Consorci Sanitari de Terrassa, 08191 Rubí, Spain; mjuanes@cst.cat (M.J.-G.); acalderonv@cst.cat (A.C.-V.); rvalls@cst.cat (R.V.-F.); clozano@cst.cat (C.L.-P.); 2 Laboratory of Surgical Neuroanatomy, Human Anatomy and Embryology Unit, Faculty of Medicine, Universitat de Barcelona, c/Casanova 143, 08036 Barcelona, Spain; 3 Management, Control and Information Analysis Unit, Hospital Universitari de Terrassa, Consorci Sanitari de Terrassa, Carretera de Torrebonica s/n, 08227 Terrassa, Spain; mgonzalezs@cst.cat; 4 Intelligence for Primary Care Research Group, Foundation University Institute for Primary Health Care Research Jordi Gol i Gurina, c/ Soler i March 6, 08242 Manresa, Spain; jvidal.cc.ics@gencat.cat; 5 Unitat de Recerca i Innovació, Gerència d’Atenció Primària i a la Comunitat de la Catalunya Central, Institut Català de la Salut, c/ Soler i March 6, 08242 Manresa, Spain

**Keywords:** COVID-19, hospital admission, death rate, vaccination, polypharmacy

## Abstract

Background: Observational studies reported worse COVID-19 evolution in relation to polypharmacy and reductions in COVID-19 hospital admissions and death in patients receiving chronic antihistamine treatment. The current profile of hospitalized patients with regard to different variants was analyzed to identify specific targets for future prospective trials. Methods: COVID-19 admissions to the Hospital of Terrassa (11 March 2020–28 August 2024 (n = 1457), from the integral Consorci Sanitari de Terrassa population (n = 167,386 people) were studied. Age, gender, the number of chronic treatments (nT), and immunization status were analyzed. Results: After 5 May 2023, 291 patients (54% females) required COVID hospitalization. Of these, 39% received >8 nT (23% receiving 5–7 nT), 70.2% were >70 years, and 93.4% survived. In total, 12% of patients admitted after 5 May 2024 were not vaccinated, while 59% received ≥4 vaccines (43% within the last 12 months). In total, 49% of admitted patients presented no previous infection (while 3% presented infection during the last year). Delta or Omicron variants would have accounted for ≥80% of admissions > 60 years compared to the first pandemic wave if no vaccines existed. Conclusions: Patients > 70 years who receive ≥5 nT, without prior COVID-19 infections, should be the priority for prevention, with updated vaccination and early treatments to reduce hospitalizations.

## 1. Introduction

After the World Health Organization (WHO) declared the end of the COVID-19 pandemic emergency on 6 May 2023, several peaks in hospitalizations in relation to different Omicron variants have occurred around the world [1].

Treatments developed during the pandemic, involving the use of corticoids [2], heparin [3], or nirmatrelvir [4] have shown positive results. In the early stages of the pandemic cross-immunity from the influenza vaccine was also described [5,6]. However, this was not confirmed in the preliminary results of our observational trial evaluating the influenza vaccine and angiotensin-converting enzyme inhibitors (ACEIs) and their influence on COVID-19 infections [7]. ACEIs have been proven to be safe [8]. Our data suggest that the influenza vaccine is associated with patients receiving polypharmacy and with comorbidity, who also show higher rates of mortality [7]. This has also been suggested by other studies evaluating frailty and COVID-19 in nursing homes [9,10].

A study in a nursing home in Yepes (Toledo, Spain) described that all residents (with about half aged over 80 years) tested positive after the first wave but that no hospital admissions or deaths occurred after treatment with antihistamines and azithromycin [11]. After applying the same treatment in primary care to 468 COVID-19 patients infected between 2020 and 2021 in that area, the hospitalization rate fell significantly compared to the official rates for Spain overall [12]. A reduction in about half of the number of hospital admissions and deaths was also observed in our institution [13], the Consorci Sanitari de Terrassa (CST), a free public integrated health care organization in the North Metropolitan Barcelona region. The CST covers seven primary health care centers, one long-term care center, and the Terrassa Hospital.

Current protocols include the use of nirmatrelvir [4], though its multiple drug interactions restrict its use. Altogether, no specific treatment is recommended in the general population when COVID-19 is diagnosed.

In order to design a prospective trial to confirm the effectiveness of antihistamine treatment in preventing hospital admissions, we studied the present profile of hospitalized patients with different COVID-19 variants to decide the target population of future interventions.

## 2. Materials and Methods

The original descriptive study of patients admitted to the hospital with COVID-19 after 1 March 2020 was approved by the Ethics Committee of the CST on 8 April 2020 (ref. 02-20-161-021), while the observational clinical trial was posted on 29 April (NCT 04367883). The inclusion of antihistamines and amantadine in relation to the reference population (n = 167,386 on 1 March 2020) was approved on 13 June 2022 (ref. 02-22-151-060) for a 3-year follow-up and posted on 17 August 2022 (https://clinicaltrials.gov/study/NCT05504057, accessed on 30 August 2024). The planning, conduct, and reporting of the study were in line with the principles of the Declaration of Helsinki.

### 2.1. Variables

Anonymized data for hospital admissions were obtained from the Data Analysis Control Department. Infection, hospital admission, and mortality rates were linked to gender, age, the number of chronic treatments (nT), the COVID-19 immunization status (having received at least one dose of a COVID-19 vaccine before the first infection (VAC or No VAC), the number of vaccines received, the number of days from the last vaccination to hospital admission and the time elapsed since the last COVID-19 infection registered in a primary care center prior to hospital admission.

### 2.2. COVID-19 Variants

**Figure 1:**
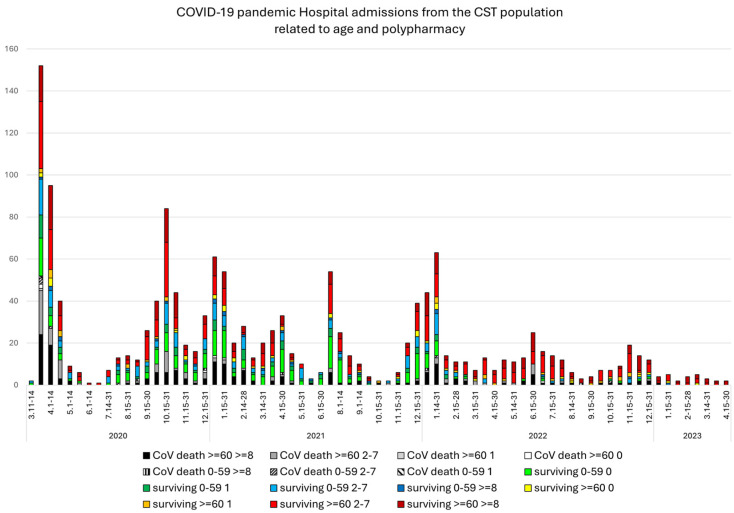
The numbers of patients who died from COVID-19 are shown in grayscale, the numbers of surviving patients aged under 60 years are illustrated in cold colors (green-blue) in relation to the number of chronic treatments received (nT: 0, 1, 2–7 or ≥8), and the numbers of surviving patients aged over 60 years are shown in warm colors (yellow-orange-red) in relation to the number of chronic treatments received (nT: 0, 1, 2–7 or ≥8).

Age (< or >60 years) and nT were reviewed during the different periods every half a month (Figure 1). The COVID-19 variants occurring in over 85% of the submitted samples during a specific period in the public databases [14] that produced different hospitalization peaks during the pandemic period were as follows:

- 1-Spanish clades [15,16]: 11 March 2020–31 May 2020;
- 20E (EU1)—Spanish clade [17]: 17 August–21 December 2020;
- 20I (Alpha): 15 March–24 April 2020;
- 21I + 21J (Delta): 19 July–6 December 2021;
- 21K (Omicron): 3 January–14 February 2022;
- 21L (Omicron): 28 March–23 May 2022;
- 22B (Omicron): 4 July–26 September 2022;
- 22E (Omicron): 18 December 2022–2 January 2023;
- 24A (Omicron): 1 January–25 March 2024;
- 24C (Omicron): 29 Julyto August 2024.

Data were included in an encrypted Excel database, which was used for the descriptive data analysis. OpenEpi web tools (Open-source Epidemiologic Statistics for Public Health) were used for statistics [18].

### 2.3. Calculation of a Theoretical Scenario without Vaccines during the Pandemic

Estimations of the hospital admission rates and deaths if no vaccines existed in relation to specific variants were based on the quantification of a 90% vaccination rate in patients aged over 60 years in our area [13]. Thus, the quantified admissions in unvaccinated patients aged over 60 years were assumed to be 10% of those that would have occurred if no vaccinations had existed. The estimated projection, multiplying the number of hospital admissions in unvaccinated patients by 10, is shown in the figure next to the absolute number of hospital admissions during that specific period.

The vaccines administered were those protocolized by the national authorities [19]. For patients aged over 70 years, the vaccination included mRNA vaccines: 3 doses of the BNT162b2 mRNA COVID-19 vaccine (Comirnaty from Pfizer-BioNTech) [20] or mRNA-1273 from Moderna [21] (for selected immunosuppressed patients) during 2021 (2 doses in the spring of 2021 and 1 booster dose in the autumn of 2021), 1 dose of Comirnaty BA4/5 in the autumn of 2022 and 1 dose of Comirnaty XBB.1.5 in the autumn of 2023. The vector vaccines ChAdOx1 nCoV-19 from AstraZeneca [22] or Ad26.COV2.S from Janssen [23] was only administered in 2021 to those aged under 70 years.

## 3. Results

### 3.1. Distribution of Hospital-Admitted Patients per Period

**Figure 2:**
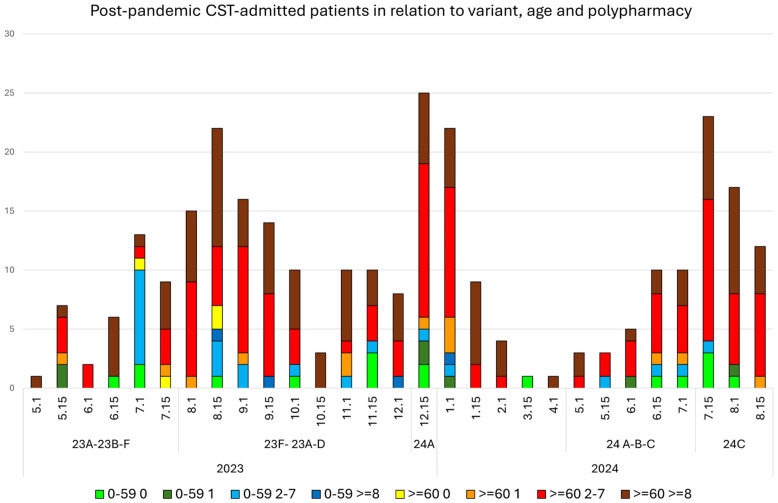
The distribution of hospital admissions every 15 days for each month in the Terrassa Hospital after 5 May 2023. Warm colors (yellow-orange-red) correspond to patients aged over 60 years, and cold colors (green-blue) correspond to patients aged under 60 years in relation to the number of chronic treatments received (nT: 0, 1, 2–7 or ≥8).

The distribution of hospital-admitted patients every half a month during the pandemic (Figure 1) and after 5 May 2023 (Figure 2) in relation to polypharmacy and age was evaluated. In total, 95.3% of patients who passed away during the pandemic period were over 60 years old; 4.3% were between 50 and 59 years old, and one was 29 years old.

**Figure 3:**
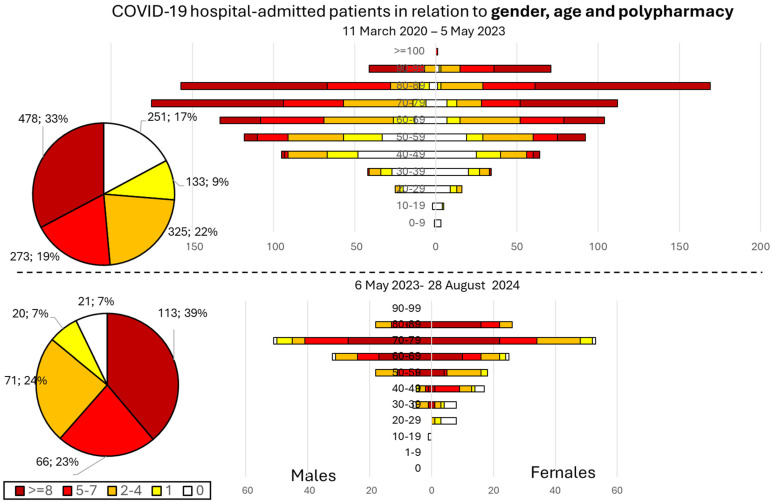
Profile of hospital-admitted patients during and after the pandemic in terms of polypharmacy (see the color scale and percentages for nT), gender, and age.

The specific distribution for gender, age, and polypharmacy is illustrated in Figure 3.

### 3.2. Profile of Hospital-Admitted Patients in Terms of Age, Gender, and Polypharmacy after 5 May 2023

During the pandemic, up to 17% of the 1460 admitted patients received no chronic treatment. That percentage diminished to 7% after 6 May 2023 (*p* = 0.00002).

After 5 May 2023, only 291 patients (54% female) required hospitalization for COVID-19. Of those, 39% had received >8 nT (23% had received 5–7 nT and 24% 2–4 nT).

Furthermore, 73% of the 18 patients who died from COVID-19 post-pandemic had received ≥10 nT, while 68% were aged ≥80 years (16% 71–79 and 11% 60–69 years old); 61.1% were males, and all had been vaccinated.

### 3.3. Hospital-Admitted Patients and Their Immunization Status

**Figure 4:**
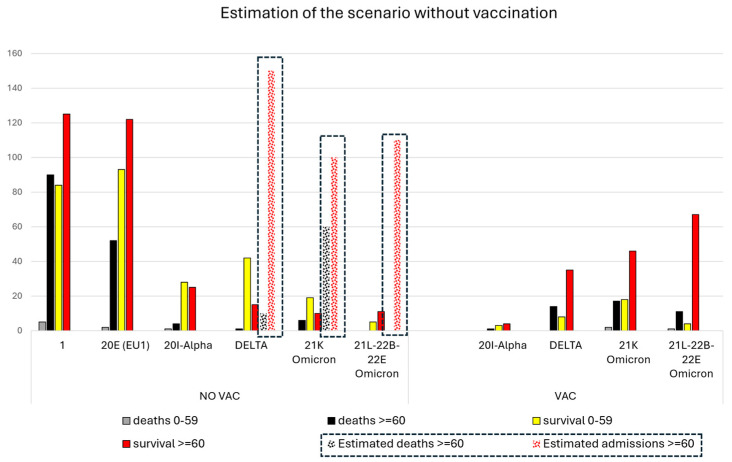
Estimations of COVID-19 deaths in patients aged over 60 years (black columns) or below 60 years (gray columns), as well as hospital admissions in those aged over or below 60 years (red and yellow, respectively) in vaccinated (VAC) and unvaccinated (NO VAC) patients for the different COVID-19 variants (predominating in >80% of the samples). Since vaccination was registered in 90% of the population aged over 60 years, we assume that the data from the unvaccinated patients correspond to 10% of the population and that estimations for a scenario in which no vaccines existed would be obtained by multiplying the data by ten. The estimations are squared. Note that the number of hospital admissions would have been comparable to that of the first wave.

Overall, hospital admissions would have been over 80% for the Delta and Omicron 21K variants when compared to the first wave of the pandemic if no vaccines existed (Figure 4).

**Figure 5:**
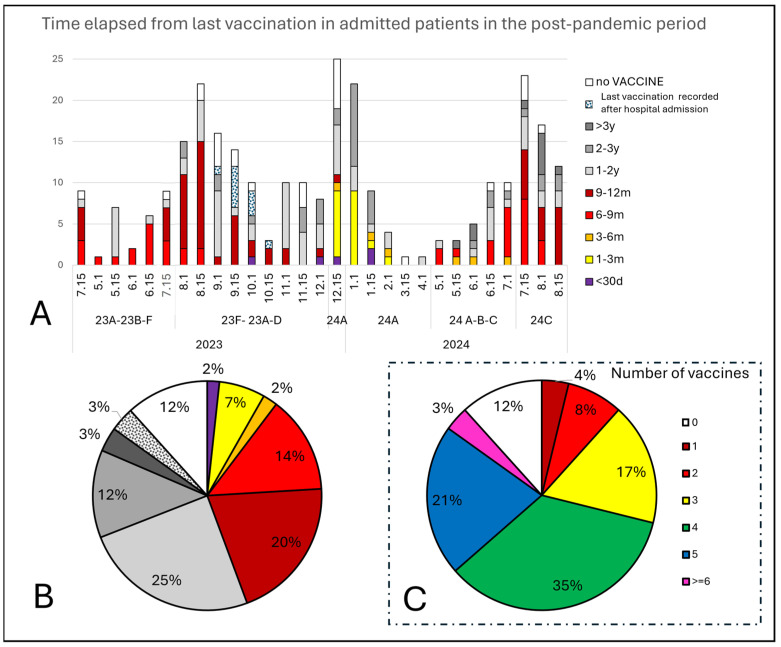
Vaccine-induced immunization (**A**,**B**). Time elapsed from the last vaccination, expressed in years (y), months (m), or days (d) per period and for each variant (**A**) and the corresponding percentage (**B**). Warm colors (red/orange/yellow) correspond to hospital admissions within 12 months after vaccination. (**C**) Percentages of hospital-admitted patients are grouped according to the number of vaccines they had received.

**Figure 6:**
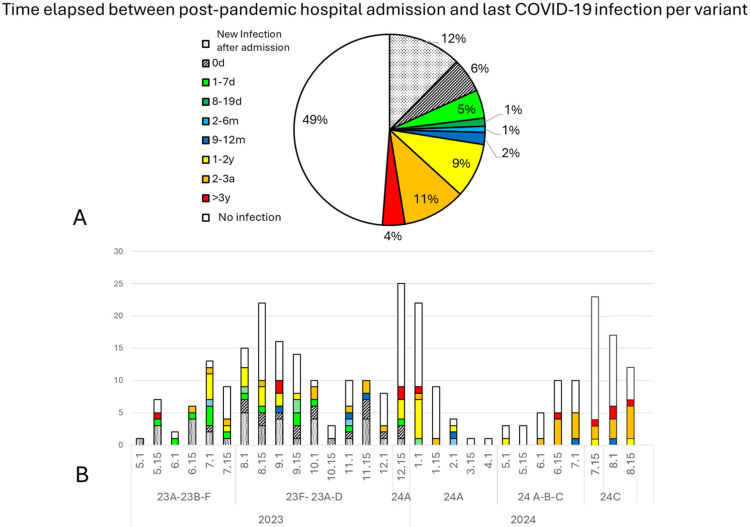
Natural immunization. (**A**) The time elapsed from the last COVID-19 infection registered in primary care to hospital admission. Some patients had a new infection recorded in primary care facilities after hospital admission (12%, shown in dotted gray). Some primary care records coincided with the day of hospital admission (0 d, shown in striped gray), while the records corresponding to between 1 and 19 days (in green) most likely reflect the symptom onset and should not be considered a new infection (12% if considered altogether). Blue colors correspond to infections within the same year (3%, 2–12 months before hospital admission), while yellow-to-red colors correspond to previous COVID-19 infections occurring between 1 and 3 years ago (24%). (**B**) Distribution of natural immunity during specific periods and for specific variants.

The post-pandemic hospital admissions in relation to immunization (the time from the last vaccine and time from the last COVID-19 infection) are presented in Figure 5 (including the percentages of patients grouped according to the numbers of vaccines they received) and Figure 6 (showing the time elapsed from the previous COVID-19 infection).

Among the patients admitted after 5 May 2023, 12% received no vaccines, while 59% had received four or more vaccines (43% within the last 12 months, and 15% were vaccinated more than 2 years ago).

Moreover, 49% of the admitted patients had no previous detection of COVID-19 infections, and 3% had experienced a COVID-19 infection in the preceding year.

## 4. Discussion

To our knowledge, this is the first study describing the COVID-19 hospitalization profile in relation to polypharmacy and the time from the last infection, as well as including common variables such as vaccination status, age, and gender. Data from the most recent COVID-19 variants of 2024 are included.

### 4.1. Estimated Hospital Admissions in a Theoretical Scenario without Vaccination

This study focused on hospital admissions since they involve the most severe forms of COVID-19 and because diagnostic tests were only available in primary care centers after the first wave (from June 2020), with the WHO recommending the end of active searches of infection in suspected cases from 2022 [24]. However, diagnostic tests were available for hospital admissions from the early stages of the COVID-19 pandemic [25] and are still being used. All patients admitted with respiratory symptoms were tested and considered to have COVID-19 if they showed bilateral interstitial pneumonia (which is rare in other illnesses) during the first wave of the pandemic.

Vaccination rates in the public primary care centers of our health consortium in early 2021 were about 90% in those aged over 60 years [13], in whom mortality from COVID-19 was still high but reduced when compared to the first wave (Figure 5). We have previously shown that hospital admissions in several age groups below 60 years were higher during the Delta variant wave compared to the first wave because vaccination was not complete in those age groups [7]. The theoretical estimations of hospital admissions in those aged over 60 years, if no vaccines existed, suggest that hospitalizations from the Delta and Omicron 21K variants would have been comparable to those of the first wave.

Mortality from COVID-19 decreased after the first wave before vaccines were available. Early treatment with corticosteroids and heparin probably reduced the incidence of cardiovascular complications, which were a common cause of mortality during the early stages of the pandemic [2,3]. Nevertheless, the estimations of mortality from the 21K variant suggests that the virulence of the Omicron variants is still high.

The number of hospital admissions is considerably reduced when compared to the early stages of the pandemic, but several peaks in hospitalization have been observed after 5 May 2023, which are comparable to those in 2022 and 2023 that were associated with the Omicron variants. The trend towards a reduction in hospital admissions observed in our area has also been described in other Mediterranean countries like Italy [1], where several peaks have also been observed during the summer. Summer peaks associated with new variants are not exclusive to Mediterranean countries. The variant BA.2.86 (23I), presenting 122 mutations (79 of them in the S gene), was quickly detected during the summer of 2023 in Denmark, Israel, South Africa, the United Kingdom, and the United States, suggesting international transmission [26]. A high number of winter peaks in hospitalization are being observed every year in Sweden, while peaks in East Asian countries like Japan are comparable to those of the first stages of the pandemic. Altogether, considering the high mutability observed in the Omicron variants in recent months [27,28], new hospitalization waves may appear in the near future.

### 4.2. Immunization: Vaccination and Previous COVID-19 Infection

Only 3% of the admitted patients had been infected with COVID-19 in the preceding year, while 49% had no previously registered COVID-19 infection. It is uncertain if the lack of registry was due to the absence of infection or to changes in the testing protocol that occurred on 23 March 2022, in which systematic diagnostic tests in symptomatic patients were no longer recommended [24]. Nevertheless, the presence of a prior infection in about a quarter of the admitted patients suggests that previous COVID-19 infections or vaccinations do not prevent new hospitalizations in frail patients.

The published official data on ‘complete vaccination’ in June 2023 in Spain indicated that more than 90% of the population aged over 60 years were vaccinated (from 92.5% for those aged 60–69 to 100% in those aged > 80 years), with most receiving mRNA vaccines during the early stages of the pandemic in 2021. All age groups received mRNA vaccines after 2022 after safety concerns for vector vaccines [19,29]. No comparison between the vaccines could be made with the data presented here since patients younger than 60 years old received both vector vaccines and mRNA vaccines in the booster doses. The high proportion of patients admitted recently, who had received four or more vaccines (59%), together with the fact that 36% had received their last dose within the last 12 months, suggests the need to update vaccines whenever possible, given the high mutability of the virus. For example, it should be noted that a high proportion of patients in December 2023 and January 2024 had received their last vaccine just 3 months before hospital admission, which included immunization against the variant XBB1.5 (23A), but not against the 23F variants occurring in the previous summer (EG.5.1) in Spain or the 23I variants [30] that have undergone numerous mutations.

### 4.3. Chronic Prescription and Polypharmacy

As we stated previously [13], chronic prescriptions were probably not affected by the existence of other private health services in the area because these are also recorded by the public health service due to cost discounts. The advanced age and high number of chronic prescriptions in hospitalized patients raise the need to explore treatment options with fewer side effects and drug interactions.

Observational experiences involving antihistamines in nursing homes [11] and primary care patients [12], as well as reductions in the hospital admissions and deaths observed in patients receiving chronic antihistamine treatment in our area [13], suggest that this treatment should be tested in prospective trials for early symptomatic treatment. Elderly individuals with polypharmacy are currently the most likely to undergo hospital admission [31], but the safety of antihistamine treatments suggests that they could also be used for symptomatic treatment in younger patients since post-COVID syndrome (which includes neurological and cognitive impairment) has been described after mild infections [32] and may appear in vaccinated individuals [33] and children [34]. Polypharmacy, as well as drug interactions, will have to be considered in future trials if the ongoing observational trials on amantadine confirm its protective role against COVID-19, as indicated by preliminary findings [35]. The design of prospective trials should also take post-COVID syndrome into account alongside hospital admission and death rates.

### 4.4. Limitations of the Study

About 90% of the population aged over 60 years belonging to the CST was vaccinated against COVID-19 by the end of 2021 [13]. However, a new primary care center (Can Roca in Terrassa City) was included in the population assigned to the CST by the end of 2022. Many of the newly assigned patients came from another public institution that used different software for clinical records. Thus, data on vaccination status might not be complete. For this reason, the estimations of the theoretical scenario without vaccines for the Omicron variants after 21K must be considered with caution, and no other estimations are shown after the wave involving the 22E variant.

The existence of another private hospital in the geographical area where COVID-19 patients may also be admitted implies that the results presented here are an underestimation of the entire impact of COVID-19.

Finally, it is uncertain if the date of infection registered in primary care after hospital admission is a late duplicated record of the previous hospital admission or corresponds to a new mild infection. However, it only affected 12% of the primary care reports on COVID-19 infections and did not affect the main conclusions of this study. Only 6% of the patients had a registered COVID-19 infection in primary care within a month prior to hospital admission, suggesting that this was the date of symptom onset. This indicates the need to improve diagnostic strategies and the early detection of hypoxemia [36] so that a hospital evaluation can be recommended before a marked deterioration in the patient and the implementation of current protocols [37].

## 5. Conclusions

COVID-19 produces periodic peaks in hospitalization comparable to some of those seen during the pandemic. Thus, updated vaccination, the prevention of infection, and the study of early treatments are still of interest and must focus on patients aged over 80 years of age who receive five or more chronic treatments and have not previously had a COVID-19 infection. Since patients without chronic treatments aged between 40 and 60 years may also need hospital admission for COVID-19 and given that post-COVID syndrome can affect the general vaccinated population and children, the prevention of infection is still recommended.

## Data Availability

Anonymized data are available from the corresponding author upon reasonable request.
